# Reprogramming of Notch1-induced acute lymphoblastic leukemia cells into pluripotent stem cells in mice

**DOI:** 10.1038/bcj.2016.57

**Published:** 2016-07-08

**Authors:** H Zhang, H Cheng, Y Wang, Y Zheng, Y Liu, K Liu, J Xu, S Hao, W Yuan, T Zhao, T Cheng

**Affiliations:** 1 State Key Laboratory of Experimental Hematology, Institute of Hematology & Blood Diseases Hospital, Chinese Academy of Medical Sciences & Peking Union Medical College, Tianjin, China; 2 Center for Stem Cell Medicine, Chinese Academy of Medical Sciences, Tianjin, China; 3 State Key Laboratory of Reproductive Biology, Institute of Zoology, Chinese Academy of Sciences, Beijing, China; 4 Department of Stem Cell and Regenerative Medicine, Peking Union Medical College, Tianjin, China

Somatic cells can be reprogrammed into induced pluripotent stem (iPS) cells using the reprogramming factors (Oct4, Sox2, Klf4 and c-Myc, also called OSKM).^1^ Cellular reprogramming and oncogenesis share many common features. The application of the iPS technology in cancers help us better understand the mechanism underlying the initiation and progression of cancer. Therefore, defining the reprogramming potential of cancer cells would provide unique opportunities to reveal epigenetic mechanisms and develop novel therapeutics for cancer. Because the reprogramming efficiency of cancer cells is paradoxically much lower than that of normal cells in general, only some handful types of cancer cells have been explored using the iPS technology.^2^ To date, in the hematopoietic system, Epstein-Barr virus (EBV)-transformed lymphoblastoid cell lines, human chronic myeloid leukemia cells, juvenile myelomonocytic leukemia cells and primary murine mixed lineage leukemia-AF9 acute myeloid leukemia cells have been successfully generated into iPS cells.^3, 4, 5, 6^ However, whether the primary malignant leukemic T cells can be reprogrammed into the iPS cells is still a mystery.

We first employed OSKM transgenic mice in which OSKM factors can be induced by doxycycline (Dox) and established the T-cell acute lymphoblastic leukemia (T-ALL) mouse model by transfecting the Lineage negative (Lin^−^) bone marrow cells from the OSKM mice with a Notch1-green fluorescent protein (GFP) retrovirus (Figure 1a). The mice developed leukemia within 2 months (Supplementary Figure S1A). The moribund mice exhibited a T-ALL phenotype (Supplementary Figures S1B-S1D). The flowchart shows the reprogramming scheme for the T-ALL cells (Figure 1b). GFP^+^ leukemia cells were sorted and plated on mouse embryonic fibroblast feeder cells. After the formation of mouse embryonic stem (ES)-like colonies, a single colony was picked up and cultured on feeder cells to produce iPS cell lines (Figure 1c). Overall, the reprogramming efficiency was very low, only approximately 0.005±0.0005% (Supplementary Figure S1E). Similarly to our previous study on acute myeloid leukemia cells.^4^ GFP was not expressed in the established leukemia iPS (L-iPS) cells (Figure 1d), indicating that the retroviral vector was silenced in L-iPS cells. The expression of pluripotency markers Oct4, Nanog and SSEA-1 was confirmed by immunofluorescence staining (Figure 1e) and qRT-PCR analyses (Supplementary Figure S2A). Genomic PCR demonstrated the presence of ectopic Notch1 and immunoglobulin heavy chain rearrangement in all tested L-iPS cells, confirming that L-iPS cells were indeed derived from T-ALL cells (Supplementary Figures S2B and S2C). Moreover, the L-iPS cell lines were predominantly diploid with the normal (40, XY) karyotype (Supplementary Figure S2D).

To further investigate the developmental potential of the L-iPS cells, we performed the teratoma assay in severe combined immunodeficiency (SCID) mice. The teratomas showed all three germ layers (Figures 1f–h). Furthermore, L-iPS cell lines were randomly selected for chimeric assessment, and two L-iPS cell lines generated eight postnatal chimeras, as chimerism reflected by coat color (Figure 1i). Notably, the chimeric mice developed recurrent leukemia within 50 days (Supplementary Figures S2E–S2H). Therefore, we were unable to test germ-line transmission using the chimeras. Taken together, we successfully established T-ALL-derived iPS cell lines even at a very low efficiency and further characterized the pluripotency of the L-iPS cells.

Interestingly, during the reprogramming process, we found two types of primary colonies, GFP^+^ and GFP^−^ colonies (Figure 2a). Both types of colonies showed typical ES-like morphology. However, only GFP^−^ colonies could be passaged to form iPS cell lines (Figure 2b), indicating an incomplete reprogrammed state of GFP^+^ colonies and a complete reprogrammed state of GFP^−^ colonies. The inhibition of Notch has been shown to greatly improve the reprogramming efficiency of both mouse and human cells^6, 7^. The retroviral-expressed Notch1 in certain leukemia cells was not sufficient to silence. This may explain at least in part why the GFP^+^ colonies could not be fully reprogrammed into iPS cells. Therefore, to understand the differences between the GFP^+^ and GFP^−^ colonies, we compared their gene expression by microarray. The gene expression profile of GFP^−^ colonies was much more similar to L-iPS cells than the GFP^+^ colonies (Supplementary Figure S3A). The gene expression trend showed that the expression of pluripotency genes, such as Nanog, Sox2, Oct4 and Sall4, were continuously increased from the leukemia cells to the L-iPS cells (Supplementary Figure S3B).

The gene expression differences between GFP^+^ and GFP^−^ colonies may provide insights into the potential barriers for reprogramming of T-ALL cells. Gene set enrichment analysis demonstrated that apoptosis, NF-κB, DOT1L, LSD1 and HDAC signature genes were significantly enriched in the GFP^+^ colonies compared with the GFP^−^ colonies (Figure 2c and Supplementary Table S1). As previous studies have reported that NF-κB served as a barrier for reprogramming.^8^ and inhibitors for apoptosis, DOT1L, LSD1 and HDAC were able to improve the reprogramming efficiency.^9, 10, 11, 12^ We wanted to know whether these signaling pathways played pivotal roles in T-ALL cell reprogramming. Therefore, we used Z-VAD-FMK, Micheliolide, EPZ004777, Tranylcypromine and Valproic acid (VPA) as inhibitors for apoptosis, NF-κB, DOT1L, LSD1 and HDAC, respectively. To ascertain whether inhibition of these pathways can improve the reprogramming of T-ALL cells, we treated leukemia cells with these inhibitors during reprogramming induction in cultures. Alkaline phosphatase staining revealed slightly higher reprogramming efficiency in individual inhibitor treated group than in dimethylsulphoxide control, except in the VPA-treated group (Figures 2d and e). Notably, we observed more than seven-fold increase in reprogramming efficiency when the cocktail of the five inhibitors was used (Figures 2d and e). In consideration that VPA had no effects on T-ALL cells reprogramming efficiency, we then used the cocktail of only four inhibitors without VPA (Figures 2d and e). The result showed that VPA had no contribution to the cocktail. Therefore, the four-inhibitor cocktail suggested a synergistic effect on reprogramming. Taken together, our results indicate that the inhibition of apoptosis, NF-κB, DOT1L and LSD1 can increase the reprogramming efficiency of T-ALL cells.

Reprogramming is a multi-step process involving extensive changes in the transcriptional and epigenetic states. Our study demonstrated that the primary mouse T-ALL cells can also be fully reprogrammed into an iPS state and revealed the barriers in the reprogramming process for T-ALL cells. NF-κB was previously showed as a barrier for reprogramming, and DOT1L served as a prominent effector of NF-κB signaling.^8^ Moreover, NF-κB pathway was highly activated in human T-ALL and inhibition of the pathway could significantly restrict the development of Notch1-induced T-ALL.^13^ suggesting that the oncogenesis of T-ALL was apparently different from the induced pluripotent reprogramming progress. Furthermore, given the fact that activation of NF-κB pathway was shown to be associated with leukemia stem cells.^14^ forced reprogramming of bulk leukemia cells did not necessarily promote the process toward the leukemia stem cell state. Therefore, further studies are certainly required to fully characterize the relationship between reprogramming and stemness, especially in the context of a hematopoietic malignant state. DOT1L inhibition could synergize with Notch inhibition to induce pluripotency at a high efficiency.^7^ Moreover, LSD1 acted as a Notch transcriptional coactivator in the context of the Notch-activation complex.^15^ Therefore, several barriers in our study were associated with chromatin-modifying enzymes, but their precise mechanisms in reprogramming remain elusive. The relationship between the chromatin modulation and the reprogramming process need to be further studied. In this work, we identified apoptosis, NF-κB, DOT1L and LSD1 pathways as barriers to T-ALL cell reprogramming by showing abortion of reprogramming and enriched their pathways in reprogramming-incompetent colonies compared to reprogramming-competent colonies. More importantly, their inhibitors were able to improve the reprogramming efficiency of T-ALL cells, and the combination increased obviously. Nevertheless, the study shows that the reprogramming of T-ALL cells to pluripotency will provide a more efficient and valuable way for the study of molecular and epigenetic events of T-cell malignancies.

## Figures and Tables

**Figure 1:**
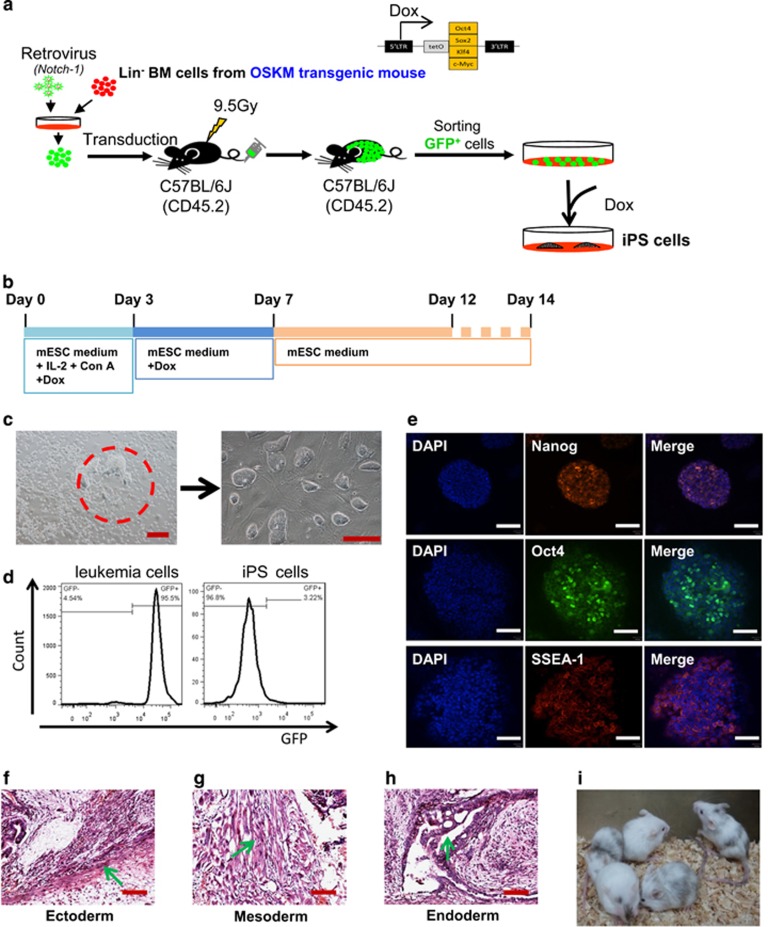
Reprogramming of T-ALL cells into iPS cells. (**a**) Experimental scheme for the reprogramming of primary T-ALL cells into iPS cells. The transduced cells were injected into lethally irradiated recipients (9.5 Gy). (**b**) Reprogramming procedure for leukemia cells. GFP^+^ leukemia cells were sorted and plated on mouse embryonic fibroblast cells at day 0. After 3 days of culture, the medium was changed to ES medium with 2 μg/ml Dox. After ES cell-like colonies appeared at day 7, the medium was replaced with ES medium until the colonies were picked up. (**c**) Representative morphology of original and passaged colonies of L-iPS cells. Scale bars, 100 μm. (**d**) FACS analysis of GFP expression in primary leukemia cells and iPS cells. (**e**) Immunofluorescence staining showing the expression of pluripotency markers (Nanog, Oct4 and SSEA-1) in L-iPS cells. Scale bars, 60 μm. (**f**–**h**) H&E staining showing teratoma from L-iPS cells containing all three germ layers. The arrows show the nerve fibers (**f**), muscular tissues (**g**) and bronchi (**h**), respectively. Scale bars, 100 μm. (**i**) Chimeric mice generated by blastocyst injection of L-iPS cells. Viable chimeric mice were shown by the coat color. H&E, hematoxylin and eosin.

**Figure 2:**
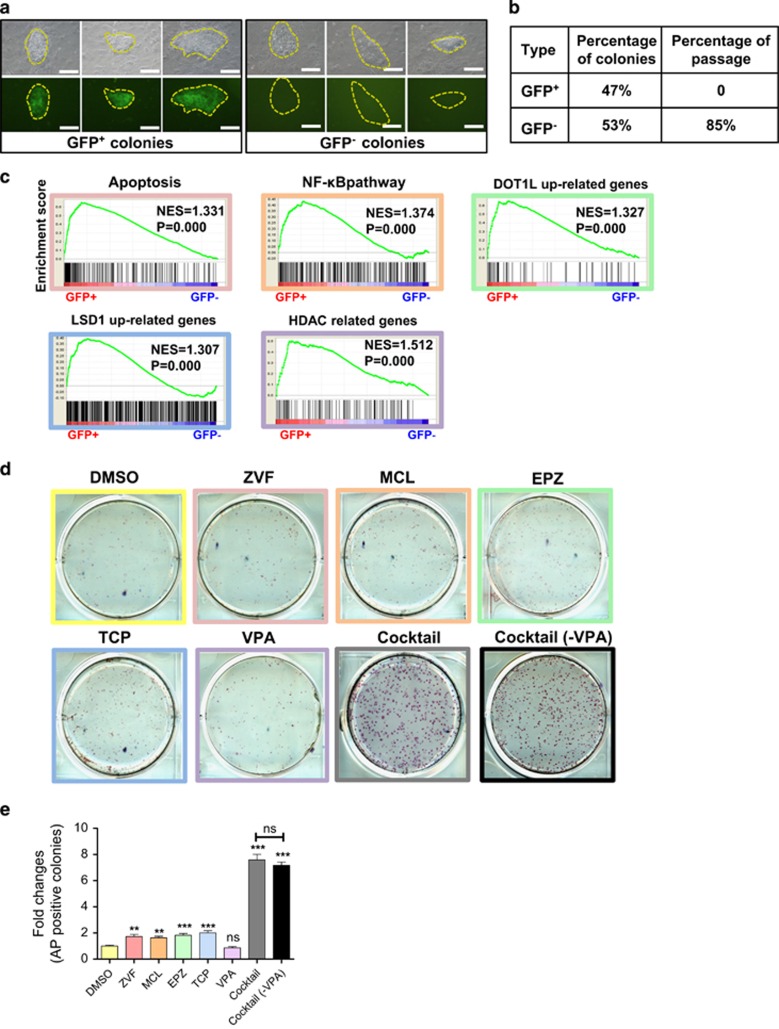
Enhancement of the reprogramming process. (**a**) Microscopy of representative images of GFP^+^ and GFP^−^ colonies in the bright field and fluorescence channel. Scale bars, 100 μm. (**b**) Statistical result showing the frequency and passaging-ability of the GFP^+^ and GFP^−^ colonies, see Figure 2a. (**c**) Gene set enrichment analysis plots revealing the expression profiles of apoptosis, NF-κB, DOTL1L, LSD1 and HDAC signature genes in GFP^+^ and GFP^−^ colonies. (**d**) Alkaline phosphatase staining of iPS colonies derived from leukemia cells cultured in systems supplemented with dimethylsulphoxide and 10 μM Z-VAD-FMK, 1 μM Micheliolide, 10 μM EPZ004777, 10 μM Tranylcypromine, 500 μM VPA, the cocktail and the cocktail without VPA. GFP^+^ leukemia cells (1 × 10^6^ cells per well) were plated on feeders in six-well plates in ES medium with Dox. (**e**) Histograms of relative reprogramming efficiency showing by AP^+^ colonies. (*n*=6, 2 independent experiments). *P<0.05, **P<0.01, ***P<0.001. Error bars indicate s.e.m.
