# Supplementary material for: Reprogramming of Notch1-induced acute lymphoblastic leukemia cells into pluripotent stem cells in mice
Source: Blood Cancer J. 2016 Jul 8;6(7):e444–. doi: 10.1038/bcj.2016.57 (PMC5030381; doi:10.1038/bcj.2016.57)

Figure S1. The phenotype of the T-ALL

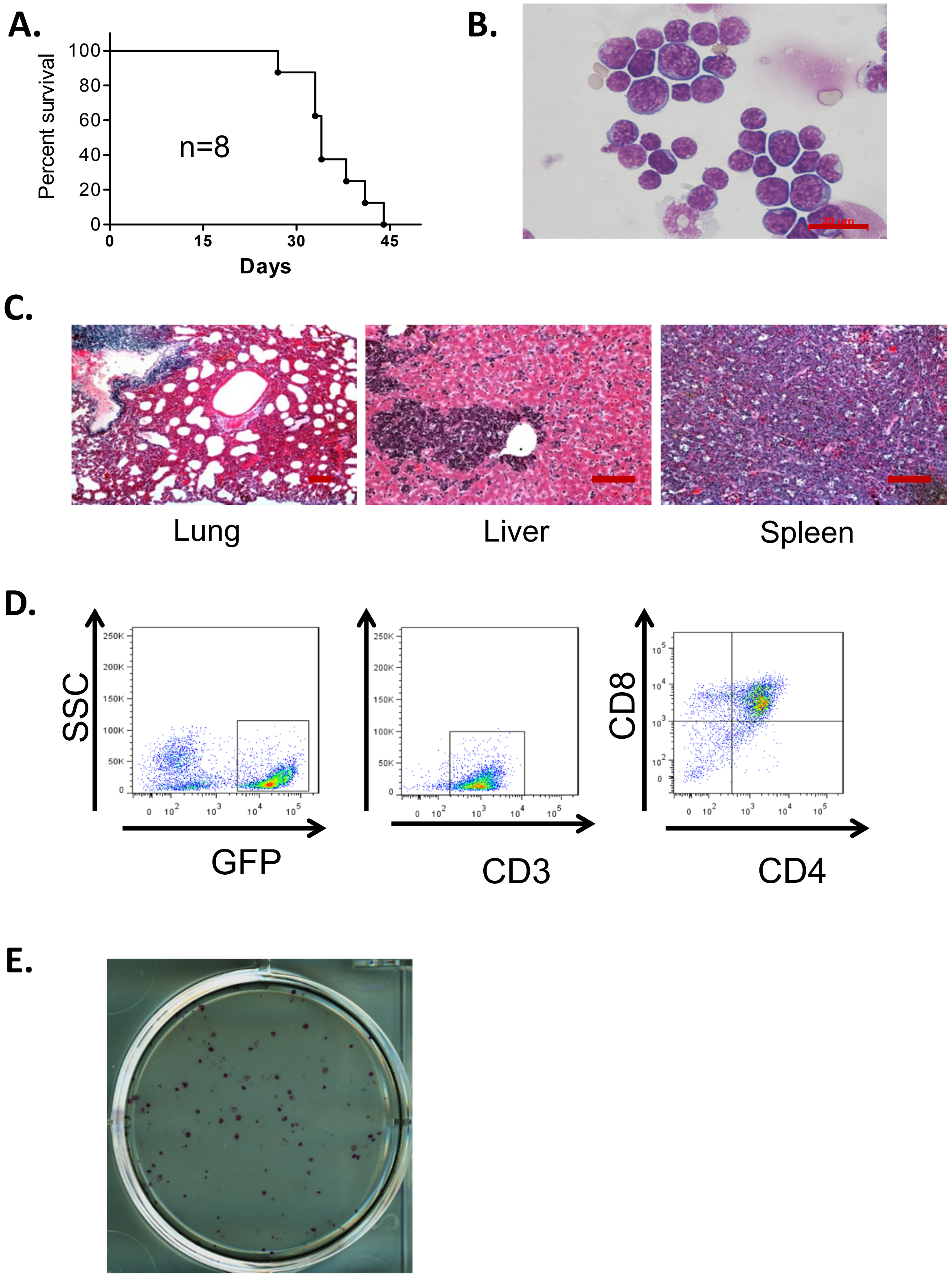

Figure S2. Characterization of the L-iPS cells

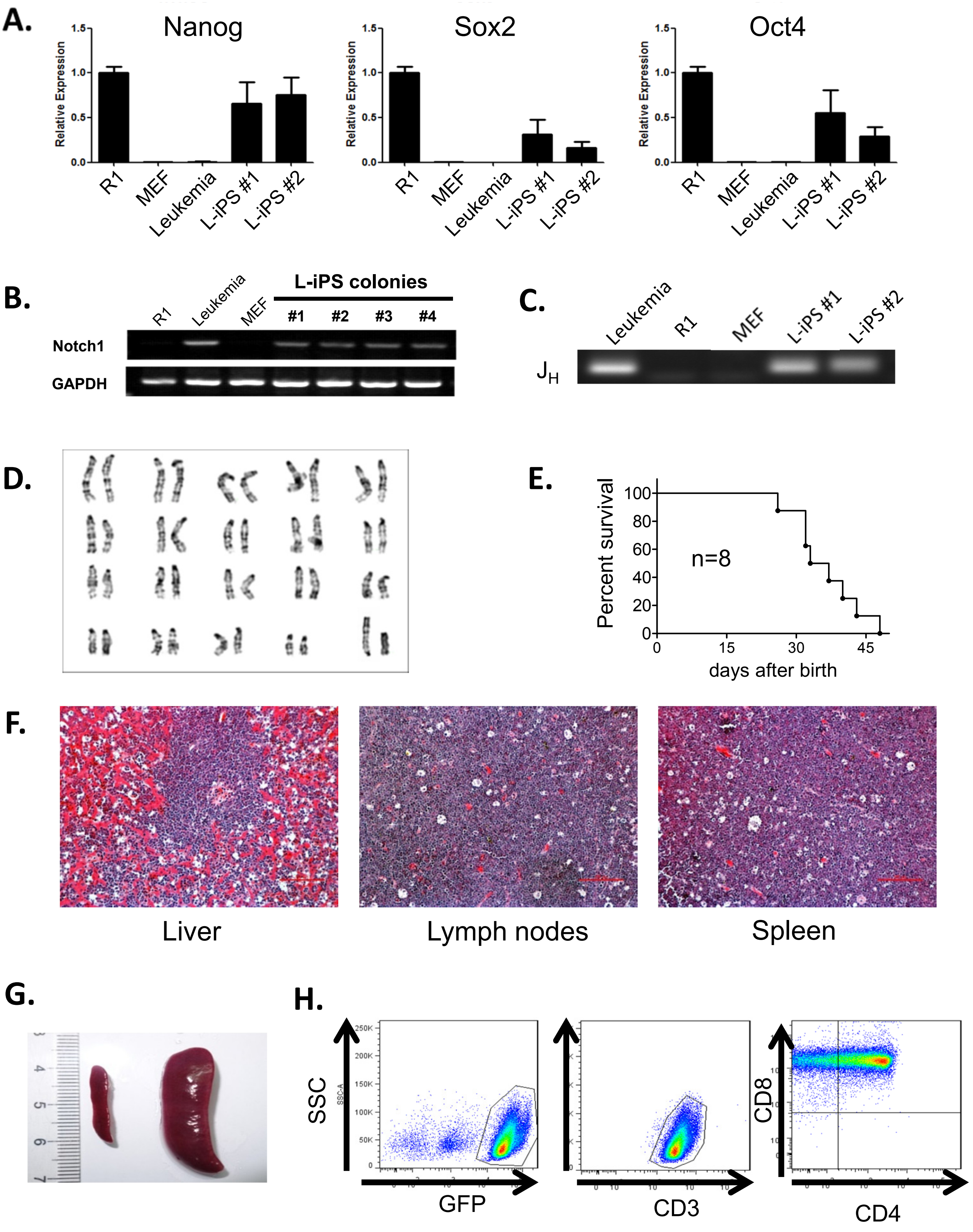

Figure S3. Gene expression analysis

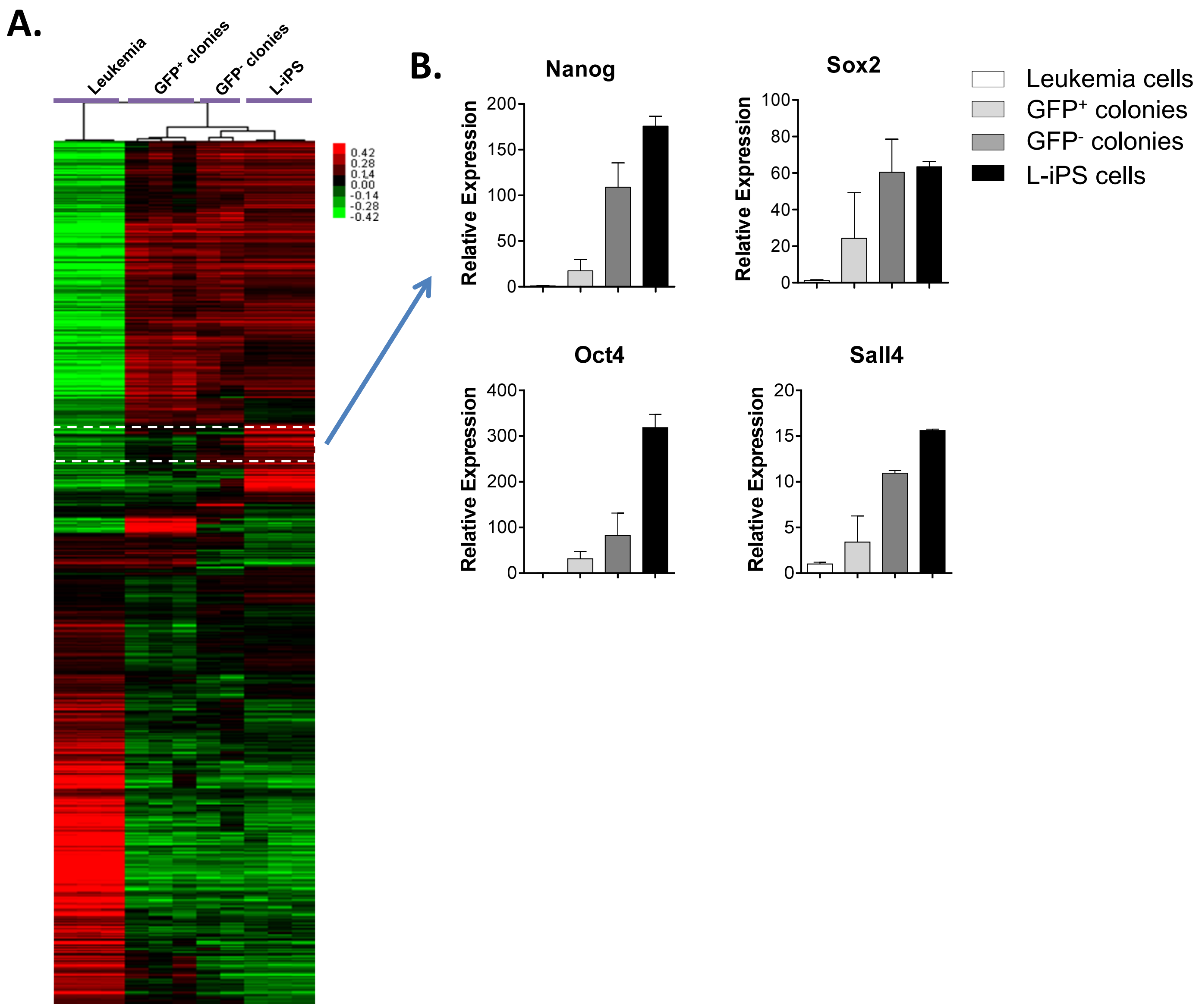

Supplement: Supplementary Information [file bcj201657x1.pdf]
